# Evaluating the effect of the COVID-19 pandemic on hypertension and diabetes care in South Korea: an interrupted time series analysis

**DOI:** 10.1186/s12889-023-16430-z

**Published:** 2023-08-12

**Authors:** Boram Sim, Sunmi Kim, Eun Woo Nam

**Affiliations:** 1 HIRA Research Institute, Health Insurance Review and Assessment Service (HIRA), Wonju, Korea; 2 Korea Pharmaceutical Information Service, Health Insurance Review and Assessment Service (HIRA), Wonju, Korea; 3 Department of Health Administration, College of Software and Digital Convergence, Yonsei University, Wonju, Korea; 4 Yonsei Global Health Center, Yonsei University, Wonju, Korea

**Keywords:** COVID-19, Pandemic, Chronic disease, Outpatient care, Access to care, Interrupted time series

## Abstract

**Background:**

Access to healthcare services is important, especially for patients with chronic diseases. We evaluated the effect of the COVID-19 pandemic on outpatient visits and medication for patients with hypertension and diabetes in South Korea.

**Methods:**

Nationwide claims data were extracted for patients with hypertension and diabetes from January 2019 to July 2020. We used an interrupted time series (ITS) analysis to evaluate the pandemic’s impact on outpatient care using the number of outpatient visits and days of medication supplied per visit. We identified the change in the continuity of care in medication, a consequence of the change in outpatient care, using the Medication Possession Ratio (MPR).

**Results:**

The number of outpatient visits for diabetes significantly declined in February 2020, when community transmission began. However, when high-intensity social distancing was relaxed in April 2020, outpatient visits for hypertension and diabetes rebounded significantly. Moreover, when the outpatient visits declined, the number of days of medication supplied per visit increased. Consequently, the average MPRs significantly increased compared to 2019, increasing the ratio of patients with appropriate medication supply (MPR ≥ 0.8).

**Conclusions:**

Outpatient visits decreased immediately when COVID-19 spread to local communities. However, the number of days of medication supplied per visit increased to compensate for the longer intervals between visits. Rather, the change in the continuity of care in medication improved; thus, the temporary decrease in outpatient visits might have had a limited negative impact on health outcomes.

**Supplementary Information:**

The online version contains supplementary material available at 10.1186/s12889-023-16430-z.

## Background

Since its discovery in Wuhan, China, in December 2019, coronavirus disease 2019 (COVID-19) has spread rapidly worldwide, prompting the World Health Organization to declare it a pandemic [1]. South Korea reported its first confirmed case of COVID-19 on January 20, 2020. The country experienced its first wave of the pandemic starting with the 31^st^ case reported on February 18 and ending in April 2020 [2]. During this period, the Korean government rapidly sorted through suspected and confirmed cases using speedy and large-scale contact tracing and diagnostic testing. In addition, it attempted to contain the spread of the virus in local communities by implementing a social distancing policy [3]. Consequently, the number of new confirmed cases soon peaked at 909 on February 29, 2020, and gradually declined thereafter [2].

However, the COVID-19 outbreak and the government's response affected many aspects of daily life. A public survey conducted during the first wave of the pandemic showed that daily life had progressively worsened with the following events: the first confirmed case in Korea (January 2020), the beginning of local transmission (February), and implementation of a stringent social distancing policy (March) [4]. Consequently, access to healthcare services was also greatly disrupted [5–7]. This calls for caution, as patients in need of healthcare services may not have had proper access to them [8].

Access to healthcare services is important, especially for patients with chronic diseases. A lack of timely healthcare service can increase the risk of emergency room visits or hospitalization due to deterioration of conditions, onset of complications, and so on [9]. Chronic diseases are responsible for 80% of all deaths in Korea. Moreover, among chronic diseases, hypertension and diabetes are the number 10th and 6th cause of death in Korea respectively, and their annual expenditure is the number one and two, imposing a considerable socioeconomic burden [10, 11]. In addition, for patients with underlying conditions, the risk of severe COVID-19 illness such as death and ICU admission is higher [12, 13]. Thus, from an epidemiological perspective of COVID-19, continuous care for chronic diseases is crucial.

Research has found changes in healthcare utilization during the COVID-19 pandemic in both Korea and abroad [14–16]. Korean studies have reported that while overall healthcare utilization decreased in 2020, healthcare utilization by patients with chronic diseases was higher than expected [17, 18]. However, these studies have some limitation. First, they are cross-sectional and do not examine the changes over time. Instead, time-series analysis can help us understand the changes in healthcare utilization over time, and reflect the trends before or during the pandemic. Second, studies have focused on changes in the volume of healthcare services, such as the number of patients or outpatient visit days. This makes it difficult to identify other aspects of continuity of care, such as medication. Patients with hypertension and diabetes need to ensure continuity of care by not only regularly visiting healthcare institutions for management of their conditions but also by taking medicine to maintain their blood pressure or keep blood sugar levels under control.

Here, we examined the impact of the COVID-19 pandemic on two aspects of outpatient care for patients with hypertension and diabetes: outpatient visits and medication. Furthermore, we identified the change in continuity of care in medication, which is the result of changes in outpatient care, before and after the pandemic.

## Methods

### Study design

First, we used an interrupted time-series (ITS) analysis to evaluate the impact of the pandemic on outpatient care. ITS analysis is useful for evaluating the impact of a policy, intervention, or event when the policy or event affects the entire population, such as the pandemic; under such circumstances, it is impossible to set a control group [19]. Furthermore, to evaluate the continuity of care in medication, we identified changes in the Medication Possession Ratio (MPR) in 2019 and 2020.

### Data source and study subjects

Nationwide claims data were extracted from the Health Insurance Review and Assessment Service (HIRA) of South Korea for patients who made outpatient visits to medical institutions with hypertension (I10–I13) or diabetes (E10-E14) as the principal or secondary diagnosis [20, 21] from January 2019 to July 2020. Owing to universal population coverage in Korea, the HIRA database covers the entire population and all healthcare providers in the country. Considering prevalence rate of chronic diseases, only patients aged 20 years or more were included [22–24]. If a claim case included both hypertension and diabetes, it was included in both groups. Among the 124,275,685 total cases, 97,906,143 and 38,330,462 cases were included in the hypertension and diabetes groups, respectively.

### Outcome variables

We used the number of outpatient visits and days of medication supplied per visit as outcome variables. Note that ITS analysis uses aggregated data collected over equally spaced time intervals. Here, the number of outpatient visits was summed up on a weekly basis. Then, considering possible variations in the number of holidays in each week, we calculated the average daily number of outpatient visits per week by dividing the total number of outpatient visits by the number of working days [25] in a week.

Moreover, the average number of days of medication supplied per visit was obtained on a weekly basis. We defined the days of medication supplied per visit as the number of days of supply of the relevant medication provided to the patient for a prescription. Thus, when determining the days of medication supplied, only claims cases that had prescriptions of antihypertensive or antihyperglycemic agents were included. To this end, we referred to the list of medicines used to assess the continuity of care for patients with hypertension and diabetes in the Quality Assessment System of NHI benefit in Korea [20, 21]; medicines for hypertension include diuretics, beta blocker, alpha blocker, angiotensin-converting-enzyme inhibitor (ACEi), angiotensin II receptor blocker (ARB), Calcium channel blockers (CCBs), medicines affecting the central nervous system, and vasodilator. Those for diabetes include biguanide, insulin secretagogue, α-Glucosidase inhibitors, insulin, incretin mimetics, thiazolidinedione (TZD), DPP-IV inhibitor and SGLT-2 inhibitor.

### Covariates

To adjust for factors that may affect healthcare service utilization, patient demographic factors (sex and age) and level of medical need (complications and comorbidities) were chosen as covariates. To adjust for the severity of hypertension or diabetes, the presence of any complications was checked using the diagnosis codes. The presence of comorbidities was expressed using the Charlson Comorbidity Index (CCI) score [26]. For this, patients' medical history, including inpatient and outpatient data, during the year prior to the study period were obtained to calculate the comorbidities. We referred to the previous studies which set one year observation period [27, 28]. Among covariates, categorical variables were described as percentages in the model.

### Medication Possession Ratio (MPR)

MPR was calculated as the total number of days of medication within the observation period divided by the total number of days in the observation period [29]. To this end, firstly, we divided patients into two groups: patients who visited the outpatient in 2019 and those in 2020, and calculated MPR by tracking each patient's medication supplies within each year. We adjusted the oversupply (MPR > 1.0) to 1.0 for each patient. We defined a patient with MPR not less than 0.8 as “an appropriate medication supply”. Lastly, the averages of the patient-specific MPR and the shares of patients with appropriate medication supply each year were obtained.

### Statistical analysis

The time interval was in weeks and a total of 83 time points were included in the analysis. There were two events of interest: the first was the occurrence of case No. 31 (week 60), who caused the spread of COVID-19 to local communities, resulting in the first wave of the pandemic in Korea. The second was the end of high-intensity social distancing (week 68), which was implemented to contain the spread of the first wave. The analysis model is as follows:

$$\mathrm{Log}({\mathrm Y}_{\mathrm t})\:=\:\mathrm{intercept}\:+\:{\mathrm\beta}_0\:\times\:{\mathrm{time}}_{\mathrm t}\:+\:{\mathrm\beta}_1\:\times\:\mathrm{event}1_{\mathrm t\:}+\:{\mathrm\beta}_2\:\times\:\mathrm{time}\;\mathrm{after}\;\mathrm{event}1_{\mathrm t}\:+\:{\mathrm\beta}_3\:\times\:\mathrm{event}2_{\mathrm t}\:+\:{\mathrm\beta}_4\:\times\:\mathrm{time}\;\mathrm{after}\;\mathrm{event}2_{\mathrm t}\:+\:{\mathrm X}_{\mathrm t}\mathrm\lambda\:+\:{\mathrm\varepsilon}_{\mathrm t}$$

where Y_t_ denotes the outcome variable at time t. Since the data for both outcome variables did not follow a normal distribution, they were log-transformed. In addition, time represents the change in time from January 2019; event1 and event2 are dummies representing the events of interest (before (0) and/or after (1) weeks 60 or 68); time after event1 and event2 are the change in time following weeks 60 or 68, respectively; X indicates covariates. β_1_ and β_3_ refer to the changes in the level of the outcome variable immediately following each event, and β_2_ and β_4_ refer to the changes in the trend of the outcome variables.

To evaluate significant differences in the continuity of care in medication, the average MPR and portion of “appropriate-medication supply” patients were compared for 2019 and 2020 using a t-test and chi-square test. All analyses were performed using SAS Enterprise Guide 7.15.

## Results

### Characteristics of patients

Table 1 presents the results of the comparison between the composition of patients in 2019 and 2020. For both hypertension and diabetes, the ratio of female patients decreased by 0.4 percentage points from 2019 to 2020, whereas the average age increased. Although the ratio of patients with complications decreased from 2019 to 2020 for both hypertension and diabetes, the proportion of diabetic patients with complications was approximately 10 times greater than that of hypertensive patients. The average CCI score decreased for both hypertension and diabetes.

**Table 1:** Characteristics of participants (Unit: Case, %)

Category	Hypertension				Diabetes			
	Jan–Dec 2019		Jan–Jul 2020		Jan–Dec 2019		Jan–Jul 2020	
Sex								
Male	29,420,241	(47.9)	17,626,093	(48.3)	12,952,902	(53.8)	7,739,673	(54.3)
Female	32,013,303	(52.1)	18,846,506	(51.7)	11,110,855	(46.2)	6,527,032	(45.8)
Age	64.6	(avg.)	64.8	(avg.)	63.3	(avg.)	63.4	(avg.)
Presence of complications								
Yes	2,724,006	( 4.4)	1,507,479	( 4.1)	10,266,954	(42.7)	6,046,326	(42.4)
No	58,709,538	(95.6)	34,965,120	(95.9)	13,796,803	(57.3)	8,220,379	(57.6)
Charlson Comorbidity Index (CCI) score								
0	12,306,129	(20.0)	8,054,053	(22.1)	5,291,583	(22.0)	3,388,911	(23.8)
1	15,717,344	(25.6)	9,430,201	(25.9)	7,304,929	(30.4)	4,360,741	(30.6)
2	13,849,572	(22.5)	8,043,250	(22.1)	5,608,304	(23.3)	3,261,841	(22.9)
3 or higher	19,560,499	(31.8)	10,945,095	(30.0)	5,858,941	(24.4)	3,255,212	(22.8)
Average Score	1.96	-	1.87	-	1.67	-	1.60	-
Total	61,433,544	(100.0)	36,472,599	(100.0)	24,063,757	(100.0)	14,266,705	(100.0)

### Impact of COVID-19 on the number of outpatient visits

**Fig. 1:**
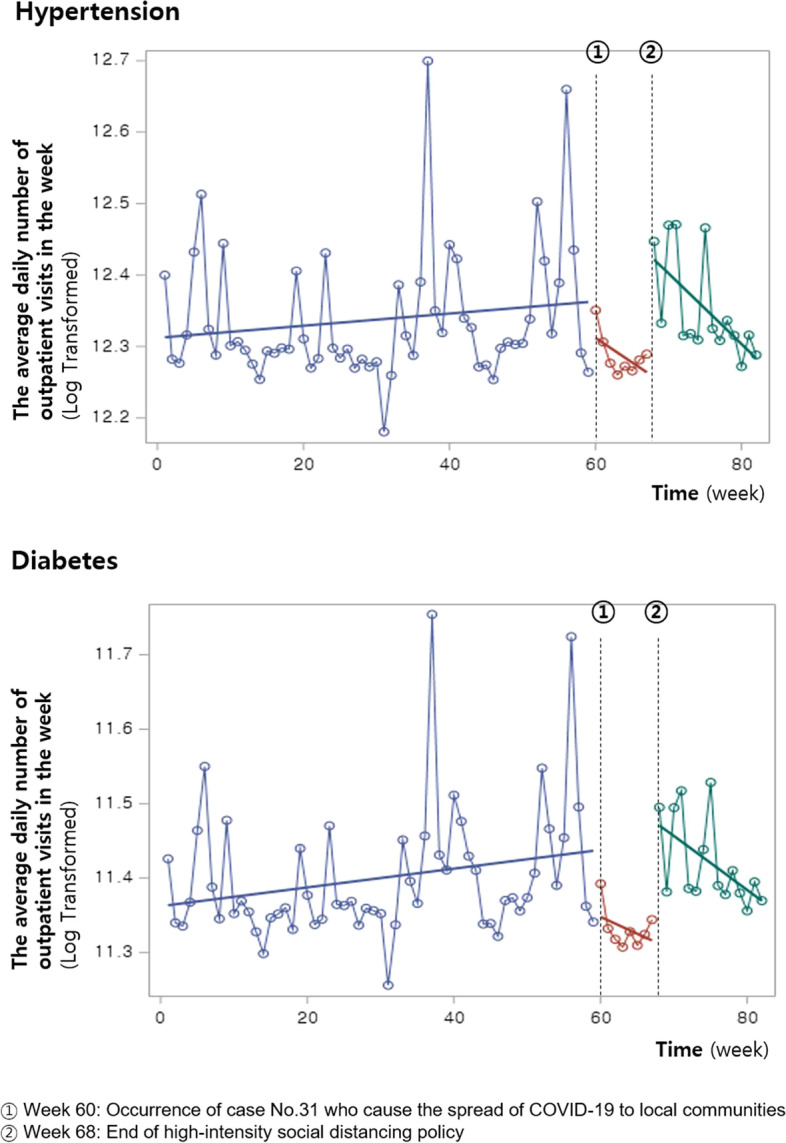
Impact of the COVID-19 pandemic on the outpatient visits for hypertension and diabetes

The average number of outpatient visits (log) for hypertension showed a gradually increasing trend starting in 2019 (Fig. 1). In February 2020, when the spread of COVID-19 to local communities began (①), the number of outpatient visits declined by 9.0%; however, this decrease was not statistically significant (Table 2). However, when high-intensity social distancing was relaxed in April 2020 (②), this number showed a statistically significant rebound of 19.0%.

**Table 2:** Impact of the COVID-19 on outpatient care for hypertension and diabetes patients

Category	Variable	Hypertension		Diabetes	
		Exp (β)	*p*-value	Exp (β)	*p*-value
Number of outpatient visits	Time (β_0_)	1.004	0.1443	1.004	0.2058
	Intervention 1 (β_1_)	0.913	0.1289	0.870	0.0161*
	Time after intervention 1 (β_2_)	0.996	0.7521	0.993	0.5325
	Intervention 2 (β_3_)	1.185	0.0057**	1.163	0.0124*
	Time after intervention 2 (β_4_)	0.994	0.6306	1.001	0.9121
Days of medication supplied per visits	Time (β_0_)	1.001	0.0083	1.001	0.0077**
	Intervention 1 (β_1_)	1.033	< 0.0001***	1.042	< 0.0001***
	Time after intervention 1 (β_2_)	0.998	0.0368	0.998	0.0492*
	Intervention 2 (β_3_)	0.995	0.3504	0.992	0.1538
	Time after intervention 2 (β_4_)	1.003	0.0063*	1.002	0.034*

^*^p < 0.05, **p < 0.01, ***p < 0.001

Regarding diabetes, the average number of outpatient visits (log) started increasing in 2019 (Fig. 1). However, this number significantly decreased by 13.0% when the spread of COVID-19 to local communities began (①) and then rebounded by 16.0% immediately following the easing of high-intensity social distancing (②) (Table 2). For both hypertension and diabetes, the trend following these two time points did not significantly change.

### Impact of COVID-19 on the days of medication supplied per visit

**Fig. 2:**
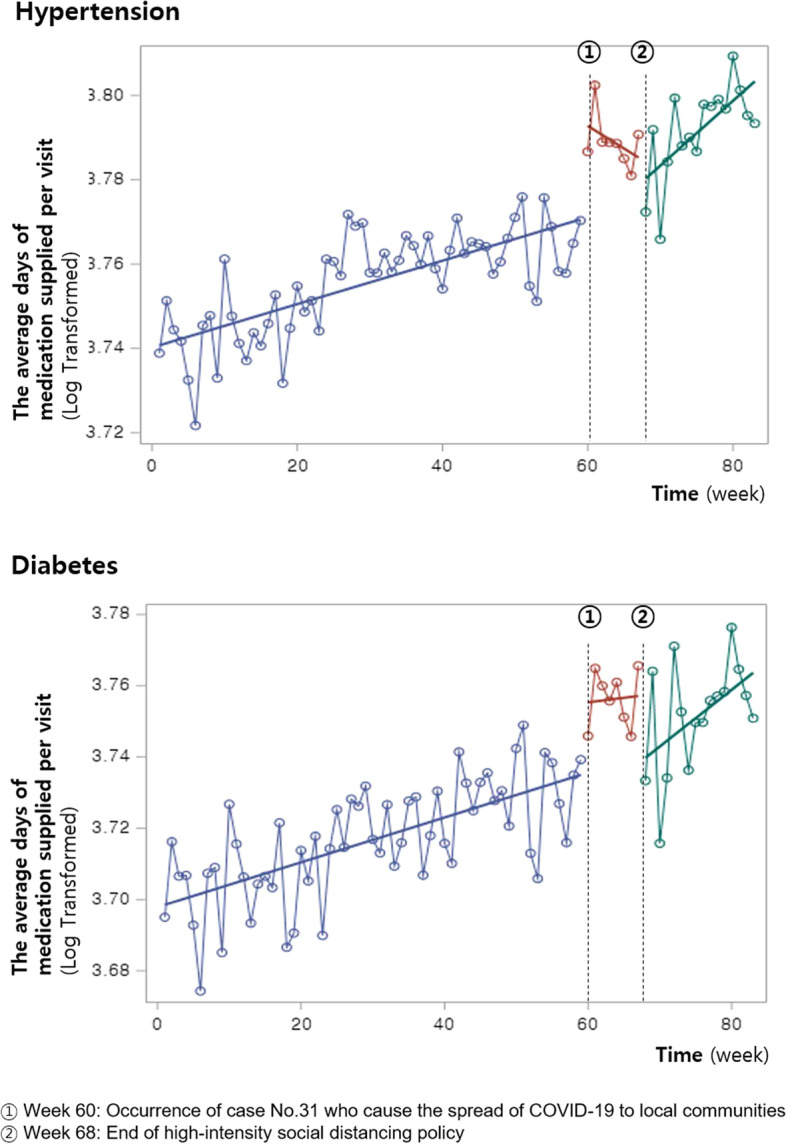
Impact of the COVID-19 pandemic on the days of medication supplied for hypertension and diabetes

The average number of days of medication supplied per visit (log) for hypertension showed a gradually increasing trend starting in 2019 (Fig. 2). When the spread of COVID-19 to local communities began in February 2020 (①), this number significantly increased by approximately 3.3% (Table 2). However, there was no significant change in the trend thereafter. At the end of the high-intensity social distancing (②), the level of days of medication supplied did not significantly change, but the trend of the slope significantly increased by 0.3%.

The average number of days of medication supplied per visit (log) for diabetes also showed a steady increase starting in 2019 (Fig. 2). When community transmission began (①), this number significantly increased by 4.2%, whereas the increasing trend of the slope slowed down by 0.2% (Table 2). When high-intensity social distancing was relaxed (②), the level of days of medication supplied did not significantly change and the trend of the slope increased significantly by 0.2%, as was the hypertension case.

### Medication Possession Ratio (MPR)

The average MPRs of hypertension and diabetes patients in 2020 were 0.82 and 0.79, respectively; compared to 2019, boththese were statistically significant increases of 0.03 (*p* < 0.0001) (Table 3). The ratio of hypertension and diabetes patients with appropriate medication supply (MPR ≥ 0.8) in 2020 was 75.0% and 70.1%, respectively; compared to 2019, these were statistically significant increases of 6.5 and 7.2 percentage points, respectively (*p* < 0.0001) (Table 3).

**Table 3:** Medication Possession Ratio (MPR) and ratio of patients with appropriate-medication supply among hypertension and diabetes patients

Category	Disease	2019 (A)	2020 (B)	Change (B-A)	*p*-value
MPR (Mean ± S.D.)	Hypertension	0.79 (± 0.28)	0.82 (± 0.23)	0.03	< 0.0001***
	Diabetes	0.76 (± 0.30)	0.79 (± 0.26)	0.034	< 0.0001***
Patients with appropriate medication supply^a^ (%, N)	Hypertension	68.5% (*N* = 4,051,064)	75.0% (*N* = 4,852,268)	6.5 percentage points	< 0.0001***
	Diabetes	62.9% (*N* = 1,314,013)	70.1% (*N* = 1,618,746)	7.2 percentage points	< 0.0001***

^*^p < 0.05, ^**^p < 0.01, ^***^p < 0.001

^a^Patients with appropriate medication supply: Patients whose MPR is not less than 0.8

## Discussion

This study demonstrated the impact of COVID-19 on outpatient care for patients with hypertension and diabetes, focusing on two aspects of outpatient visits and medication. We found that the number of outpatient visits by patients decreased immediately when COVID-19 spread to local communities, in line with results observed in other countries during the pandemic [5, 15]. The most noteworthy reason for this reduction is the patients' fear of contracting COVID-19 [30]. According to the Medical Service Experience Survey of Korea, 15.6% of respondents in the first half of 2020 answered that they felt infection-related anxiety while using medical services; this is more than a two-fold increase year-over-year from the same period in 2019 [31]. A public perception survey conducted in 2020 also showed that 73.2% of the respondents had avoided using healthcare [32]. Patients seem to have made decisions on using healthcare services by weighing the risks and benefits of doing so during that period [33]. Here, we found that the number of outpatient visits by diabetes patients significantly decreased when COVID-19 began spreading to local communities; however, no significant differences were observed for hypertension patients. This is maybe because the ratio of diabetes patients with complications is higher than that of hypertension patients; hence, the former perceived the risk of COVID-19 infection as a greater threat. Similar to our study, a study reported that people with diabetes complications were more concerned about the infections of COVID-19 than those without complications [34]. In Korea, it is reported that 84.7% of patients with type 2 diabetes experience chronic complications caused by diabetes [35]; consequently, diabetes and complications caused by diabetes are the sixth-highest cause of death. Therefore, patients with diabetes may have a greater burden of disease and perceive more the risk of COVID-19 infection.

These perceptions may have been escalated by the government's pandemic-related policies, such as social distancing [30]. We found that the number of outpatient visits, which suppressed when the high-intensity social distancing policy was enforced, rebounded significantly once the policy ended. This suggests that although this policy contributed positively to curbing the spread of COVID-19, it may have negatively affected the disease management of non-COVID-19 patients [36, 37]. In response, the Korean government took necessary measures to ensure that patients continue to receive their routine care. Representatively, there are the temporary approval of teleconsultation and designation of "National Safe Hospitals". In the National Safe Hospitals, patients with respiratory conditions are treated at an independent area to prevent contact with other patients. However, these measures failed to offset the reduction in healthcare utilization in the end. This maybe because of low awareness of policies. Especially, this is because telemedicine was not implemented in Korea before the pandemic due to many controversies over patient safety and responsibility matter [38, 39]. A previous study indicated that the teleconsultation rate in Korea is very low compared to other countries due to low awareness of teleconsultation of both doctors and patients [40]. As a result, teleconsultation accounted for a small amount of the total medical use. In this study, among the claim cases of hypertension and diabetes from 2020, very few (about 0.45% and 0.52%, respectively) were teleconsultation cases. Nevertheless, teleconsultation, which was temporarily approved during the pandemic, served as an opportunity for both patients and doctors to experience the benefits of non-face-to-face care, resulting in continuing its use even after the COVID-19 crisis ended. Once the teleconsultation become a routine way of receiving treatment, it would be more effectively used in the next health crisis. However, further research is still needed on the causes of disruptions in healthcare services and required measures.

Research from the perspective of healthcare service supply also shows that relatively low-priority healthcare services for non-COVID-19 patients were postponed or canceled due to the surge in COVID-19 [41]. However, Korea may have experienced minimal impact in this regard because its healthcare system was not overwhelmed by the COVID-19 pandemic [42]. Nevertheless, the interval between outpatient visits may have increased because of changes in doctors’ practice. For example, doctors were wary of patients becoming infected with COVID-19 while visiting their healthcare institutions [9]. In an unprecedented situation such as the pandemic, doctors took the flexible approach of providing long-term prescriptions and postponing patients' subsequently scheduled outpatient visits. We also found a significant increase in the number of days of medication supplied per visit while the number of outpatient visits were decreasing. Other countries show similar shifts in approach, which is a strategy recommended at the national level [43, 44].

Furthermore, these changes in healthcare utilization may vary depending on the risk perception of COVID-19 infection. The subgroup analysis results by age and region in Supplementary Tables shows that the reduction in the number of outpatient visits and increase in the days of medication supplied per visit was greater for the high-risk groups (i.e., patients aged 65 years or older), and patients in the Daegu and Gyeongbuk regions, which were the epicenter of the first wave of the COVID-19 pandemic in Korea. In line with research [7, 43], those at higher risk of COVID-19 showed more flexible responses to healthcare utilization from the perspective of both supply and demand.

Finally, this study found continuity of care in medication increased significantly from 2019 to 2020. This may be because the aforementioned long-term prescription strategy, in addition to the increasing number of days of medication supplied per visit. Studies have also reported improvements in medication adherence during the COVID-19 pandemic [45, 46]. Thus, this study indicates that patients with hypertension and diabetes may have continued their medication during the pandemic; the temporary decrease in outpatient visits may have had a limited negative impact on health outcomes. Note that MPR was determined from administrative data alone and may not be fully representative of whether the patient actually took the medication; still, possession of a sufficient amount of medication carries an important meaning as it is the first step in ensuring patients' ability to continue their medication [47]. Further research from a long-term perspective is necessary to determine how the delay in regular visits to the doctor or lab tests influenced disease management, and whether longer-term prescriptions were an effective strategy.

This study has several limitations. First, we analyzed data up to July 2020 and focused on changes in healthcare utilization during the first wave of the COVID-19 pandemic in Korea (February 2020 to April 2020). Thus, our work does not reflect other COVID-19 waves, such as the spread of the Omicron variant, or changes in the pandemic policy, such as the stepwise return to normal life that followed our study period. Nevertheless, note that the first wave of the pandemic was the period where other factors may have had the least effect, such as pandemic-policy fatigue. Hence, it may be the period that most clearly shows the impact of COVID-19. Second, since this study aimed to investigate average changes in outpatient care, the results may differ from those at the individual level. For example, we cannot rule out that individual patients missed opportunities to improve their health by failing to use healthcare services in a timely manner. Nevertheless, our work is meaningful in that it examined changes in the services provided (medication supplied) as well as outpatient visits using nationally representative data. Lastly, this study focused on the short-term impact in the early stages of COVID-19, so there was a limit to measuring the long-term health outcomes such as complications, hospitalization, and even death; thus, further study is needed to look at the relationship the continuity of care and health outcomes during the COVID-19. Nevertheless, given that a previous study showed better health outcomes in patients with keeping the continuity of care even during COVID-19 [48] and other studies that indicated worsen health outcomes in patient with lower MPR [49, 50], the MPR measured in our study is sufficient as an intermediate health outcome.

## Conclusions

The finding from the study revealed that the number of outpatient visits and the days of medication supplied per visits fluctuated when COVID-19 spread to local communities. However, this study suggests that patients may have continued their medication during the pandemic despites the temporary decrease in outpatient visits. Nevertheless, health crisis such as the COVID-19 pandemic affects healthcare utilization by patients with chronic diseases. Consequently, we need corresponding response measures to emerging and re-emerging infectious diseases. First, the government needs to implement transparent and reliable risk communication strategies to prevent individuals from experiencing excessive anxiety during health crises. Second, a change in the healthcare delivery system is also required to provide healthcare services to patients in a timely manner. In particular, if enforcing mobility restriction policies such as social distancing, patients should be provided with alternatives other than face-to-face care. In Korea, a nationwide pilot of telemedicine is now ongoing, and in order for it to be used effectively in the next health crisis, discussions should continue on how to ensure the quality of care and safety.

### Supplementary Information

**Additional file 1:** **Table S1.** Impact of the COVID-19 on the number of outpatient visits for hypertension and diabetes patients by age groups and regions. **Table S2.** Impact of the COVID-19 on the days of medication supplied per visits for hypertension and diabetes patients by age groups and regions.

## Data Availability

The data that support the findings of this study are available from the Health Insurance Review and Assessment Service but restrictions apply to the availability of these data, which were used under license for the current study, and so are not publicly available. Data are however available from the first author (Boram Sim, simbr12@hira.or.kr) upon reasonable request and with permission of the Health Insurance Review and Assessment Service.

## Abbreviations

CCI: Charlson Comorbidity Index
COVID-19: Coronavirus disease 2019
HIRA: Health Insurance Review and Assessment Service
ITS: Interrupted time series
MPR: Medication Possession Ratio
