# Supplementary material for: Evaluating the effect of the COVID-19 pandemic on hypertension and diabetes care in South Korea: an interrupted time series analysis
Source: BMC Public Health. 2023 Aug 12;23:1538. doi: 10.1186/s12889-023-16430-z (PMC10422749; doi:10.1186/s12889-023-16430-z)
Supplement: Supplementary file 1 — Additional file 1: Table S1. Impact of the COVID-19 on the number of outpatient visits for hypertension and diabetes patients by age groups and regions. Table S2. Impact of the COVID-19 on the days of medication supplied per visits for hypertension and diabetes patients by age groups and regions. [file 12889_2023_16430_MOESM1_ESM.docx]

Supplementary Tables

Supplementary Table 1. Impact of the COVID-19 on the number of outpatient visits for hypertension and diabetes patients by age groups and regions

| Disease  groups | Sub-groups | Time(β_0_) | | Intervention1(β_1_) | | Time after  Intervention1(β_2_) | | Intervention2(β_3_) | | Time after  Intervention2(β_4_) | |
| --- | --- | --- | --- | --- | --- | --- | --- | --- | --- | --- | --- |
|  |  | Exp(β) | p-value | Exp(β) | p-value | Exp(β) | p-value | Exp(β) | p-value | Exp(β) | p-value |
| Hyper-tension | Ages |  |  |  |  |  |  |  |  |  |  |
|  | 20-39 years | 1.00 | 0.2899 | 0.876 | 0.0567 | 0.995 | 0.6825 | 1.134 | 0.0775 | 0.999 | 0.9383 |
|  | 40-64 years | 1.00 | 0.2736 | 0.889 | 0.0665 | 0.995 | 0.6900 | 1.144 | 0.0361* | 0.998 | 0.8487 |
|  | 65 years and over | 1.00 | 0.6713 | 0.930 | 0.2488 | 0.994 | 0.5982 | 1.220 | 0.0014** | 0.996 | 0.7605 |
|  | Regions |  |  |  |  |  |  |  |  |  |  |
|  | Capital regions | 1.00 | 0.4357 | 0.888 | 0.0435* | 1.001 | 0.9286 | 1.162 | 0.0129* | 0.991 | 0.4668 |
|  | Daegu-Gyeongbuk regions | 1.00 | 0.4606 | 0.951 | 0.5147 | 0.993 | 0.6447 | 1.226 | 0.0068* | 0.995 | 0.7554 |
|  | Others | 1.00 | 0.4344 | 0.949 | 0.4181 | 0.987 | 0.2990 | 1.209 | 0.0042* | 1.003 | 0.8063 |
| Diabetes | Ages |  |  |  |  |  |  |  |  |  |  |
|  | 20-39 years | 1.00 | 0.6449 | 0.873 | 0.0300* | 1.000 | 0.9811 | 1.115 | 0.0886 | 0.998 | 0.8841 |
|  | 40-64 years | 1.00 | 0.5262 | 0.847 | 0.0060* | 0.998 | 0.8535 | 1.142 | 0.0263* | 0.999 | 0.9160 |
|  | 65 years and over | 1.00 | 0.0842 | 0.888 | 0.0530 | 0.996 | 0.7191 | 1.165 | 0.0136* | 0.996 | 0.7478 |
|  | Regions |  |  |  |  |  |  |  |  |  |  |
|  | Capital regions | 1.00 | 0.1052 | 0.851 | 0.0029* | 0.998 | 0.8592 | 1.141 | 0.0197* | 0.997 | 0.7943 |
|  | Daegu-Gyeongbuk regions | 1.00 | 0.2704 | 0.900 | 0.1592 | 0.986 | 0.2945 | 1.234 | 0.0065* | 1.008 | 0.6038 |
|  | Others | 1.00 | 0.8094 | 0.893 | 0.0704 | 0.991 | 0.4198 | 1.183 | 0.0090* | 1.003 | 0.8053 |

* p < 0.05, ** p < 0.01, *** p < 0.001

Supplementary Table 2. Impact of the COVID-19 on the days of medication supplied per visits for hypertension and diabetes patients by age groups and regions

| Disease  groups | Sub-groups | Time(β_0_) | | Intervention1(β_1_) | | Time after  Intervention1(β_2_) | | Intervention2(β_3_) | | Time after  Intervention2(β_4_) | |
| --- | --- | --- | --- | --- | --- | --- | --- | --- | --- | --- | --- |
|  |  | Exp(β) | p-value | Exp(β) | p-value | Exp(β) | p-value | Exp(β) | p-value | Exp(β) | p-value |
| Hyper-tension | Ages |  |  |  |  |  |  |  |  |  |  |
|  | 20-39 years | 1.00 | 0.0002** | 1.028 | 0.0026** | 0.996 | 0.0162 | 0.993 | 0.4600 | 1.006 | 0.0023* |
|  | 40-64 years | 1.00 | 0.0005** | 1.030 | <.0001*** | 0.997 | 0.0099* | 0.997 | 0.5893 | 1.004 | 0.0022* |
|  | 65 years and over | 1.00 | 0.0026* | 1.034 | <.0001*** | 0.998 | 0.1305 | 0.992 | 0.1436 | 1.003 | 0.0253* |
|  | Regions |  |  |  |  |  |  |  |  |  |  |
|  | Capital regions | 1.00 | 0.0229* | 1.031 | <.0001*** | 0.998 | 0.0300* | 0.996 | 0.4055 | 1.003 | 0.0035* |
|  | Daegu-Gyeongbuk regions | 1.00 | 0.0002** | 1.040 | <.0001*** | 0.997 | 0.0047* | 0.994 | 0.2928 | 1.004 | 0.0027* |
|  | Others | 1.00 | 0.0001** | 1.033 | <.0001*** | 0.998 | 0.0451* | 0.995 | 0.4031 | 1.003 | 0.0098* |
| Diabetes | Ages |  |  |  |  |  |  |  |  |  |  |
|  | 20-39 years | 1.00 | <.0001*** | 1.042 | <.0001*** | 0.995 | 0.0040* | 0.992 | 0.3180 | 1.005 | 0.0058* |
|  | 40-64 years | 1.00 | 0.0596 | 1.038 | <.0001*** | 0.998 | 0.0284* | 0.989 | 0.0631 | 1.003 | 0.0118* |
|  | 65 years and over | 1.00 | 0.0066* | 1.047 | <.0001*** | 0.998 | 0.0549 | 0.996 | 0.4922 | 1.003 | 0.0440* |
|  | Regions |  |  |  |  |  |  |  |  |  |  |
|  | Capital regions | 1.00 | 0.0498* | 1.033 | <.0001*** | 0.999 | 0.4572 | 0.990 | 0.0900 | 1.001 | 0.2959 |
|  | Daegu-Gyeongbuk regions | 1.00 | 0.0421 | 1.060 | <.0001*** | 0.995 | <.0001*** | 0.996 | 0.5811 | 1.006 | 0.0001** |
|  | Others | 1.00 | <.0001*** | 1.045 | <.0001*** | 0.997 | 0.0061* | 0.994 | 0.3532 | 1.003 | 0.0056* |

* p < 0.05, ** p < 0.01, *** p < 0.001
